# An Encapsulation of Gene Signatures for Hepatocellular Carcinoma, MicroRNA-132 Predicted Target Genes and the Corresponding Overlaps

**DOI:** 10.1371/journal.pone.0159498

**Published:** 2016-07-28

**Authors:** Xin Zhang, Wei Tang, Gang Chen, Fanghui Ren, Haiwei Liang, Yiwu Dang, Minhua Rong

**Affiliations:** 1 Research Department, Affiliated Cancer Hospital, Guangxi Medical University, 71 Hedi Road, Nanning, Guangxi Zhuang Autonomous Region, P. R. China; 2 Department of Breast Surgery, Affiliated Cancer Hospital, Guangxi Medical University, 71 Hedi Road, Nanning, Guangxi Zhuang Autonomous Region, P. R. China; 3 Department of Pathology, First Affiliated Hospital of Guangxi Medical University, 6 Shuangyong Road, Nanning, Guangxi Zhuang Autonomous Region, P. R. China; Taipei Medicine University, TAIWAN

## Abstract

**Objectives:**

Previous studies have demonstrated that microRNA-132 plays a vital part in and is actively associated with several cancers, with its tumor-suppressive role in hepatocellular carcinoma confirmed. The current study employed multiple bioinformatics techniques to establish gene signatures for hepatocellular carcinoma, microRNA-132 predicted target genes and the corresponding overlaps.

**Methods:**

Various assays were performed to explore the role and cellular functions of miR-132 in HCC and a successive panel of tasks was completed, including NLP analysis, miR-132 target genes prediction, comprehensive analyses (gene ontology analysis, pathway analysis, network analysis and connectivity analysis), and analytical integration. Later, HCC-related and miR-132-related potential targets, pathways, networks and highlighted hub genes were revealed as well as those of the overlapped section.

**Results:**

MiR-132 was effective in both impeding cell growth and boosting apoptosis in HCC cell lines. A total of fifty-nine genes were obtained from the analytical integration, which were considered to be both HCC- and miR-132-related. Moreover, four specific pathways were unveiled in the network analysis of the overlaps, i.e. adherens junction, VEGF signaling pathway, neurotrophin signaling pathway, and MAPK signaling pathway.

**Conclusions:**

The tumor-suppressive role of miR-132 in HCC has been further confirmed by *in vitro* experiments. Gene signatures in the study identified the potential molecular mechanisms of HCC, miR-132 and their established associations, which might be effective for diagnosis, individualized treatments and prognosis of HCC patients. However, combined detections of miR-132 with other bio-indicators in clinical practice and further *in vitro* experiments are needed.

## 1. Introduction

Hepatocellular carcinoma (HCC) is among the most common cancers and ranks as the third most frequent cause of cancer-related deaths globally.[1] Nevertheless, diagnosis of HCC is often made at an advanced stage and drug resistance and recurrence are often observed in HCC, leaving poor prognosis for HCC patients.[2, 3] Thus, there are urgent demands that novel diagnostic and prognostic biomarkers for HCC should be discovered and that a clearer map of molecular mechanisms of HCC should be drawn.

Gene signatures, which are considered auspicious in diagnosing and prognosis-predicting for HCC, can furnish us with molecular bases, regulatory pathways and mediating networks of HCC pathogenesis, thus leading to an improved route for earlier detection and more personalized treatment strategies for HCC.[4]

MicroRNAs, or miRNAs in short, are an abundant class of small non-coding RNA molecules, acting as regulators in nearly one third of protein-coding genes at post-transcriptional level.[5] During the last decade, miRNAs have been proved to be active and crucial in human carcinogenesis via mediating protein expressions. [6]

MiR-132, one of the most vigorously studied miRNAs, is located in chromosome 17p13.3, which has exhibited connections with a variety of malignancies such as breast cancer[7], colorectal cancer[8], gastric cancer[9], glioma[10], osteosarcoma[11], pancreatic cancer[12], and prostate cancer[13]. Initially, Wei, et al. [14] explored the potential role of miR-132 may play in HBV-mediated hepatocarcinogenesis, and demonstrated the down-regulation of miR-132 in HBV-related HCC with a cohort of 20 patients. Later on, in our previous study, we have validated the down-regulation of miR-132 in HCC with a larger cohort of 95 patients and confirmed its tumor suppressive role in HCC on the basis of determined relationships between miR-132 and several clinical/pathological indicators and recurrence data in HCC patients *(Xin Zhang*, *et al*. *Down-regulation of MicroRNA-132 Indicates Progression in Hepatocellular Carcinoma*: *A Clinical Perspective*. *Experimental and Therapeutic Medicine*. *In Press*.*)*. Recently, Liu, et al[15] has conducted a series of tasks, including comparing expressions of miR-132 between HCC and adjacent non-cancerous liver tissue, as well as in several cell lines, exploring cellular functions of miR-132 in HCC via multiple assays, confirming tumor suppressive role of miR-132 in HCC with nude mouse model and establishing PIK3R3 as a new target gene of miR-132. However, the overview for molecular mechanisms on the decrease of miR-132 in HCC remains obscure in spite of the previous researches. Given the complexity of multi-level regulatory systems in oncogenesis, a comprehensive and systematic analysis of miR-132 signatures in HCC is pressingly thirsted, which will not only feature the potential molecular mechanisms of miR-132 in HCC but also provide insights into diagnostic methods, therapeutic strategies and prognostic assessments of HCC.

In the current study, *in vitro* experiments were conducted to further verify the down-regulation of miR-132 and to assess its cellular functions in HCC with a quadrupled scale of four HCC cell lines compared to the study led by Liu, et al.[15]. More importantly, we performed a successive panel of data mining and screening, target genes prediction, comprehensive analyses, which included gene ontology (GO) analysis, pathway analysis and network analysis, and later analytic integration in an attempt to offer a comprehensive and systematic panorama on the expression of potential target genes of miR-132 related to carcinogenesis, metastasis, prognosis, recurrence, survival and drug-resistance (sorafenib and bevacizumab) in HCC.

## 2. Materials and Methods

**Fig 1:**
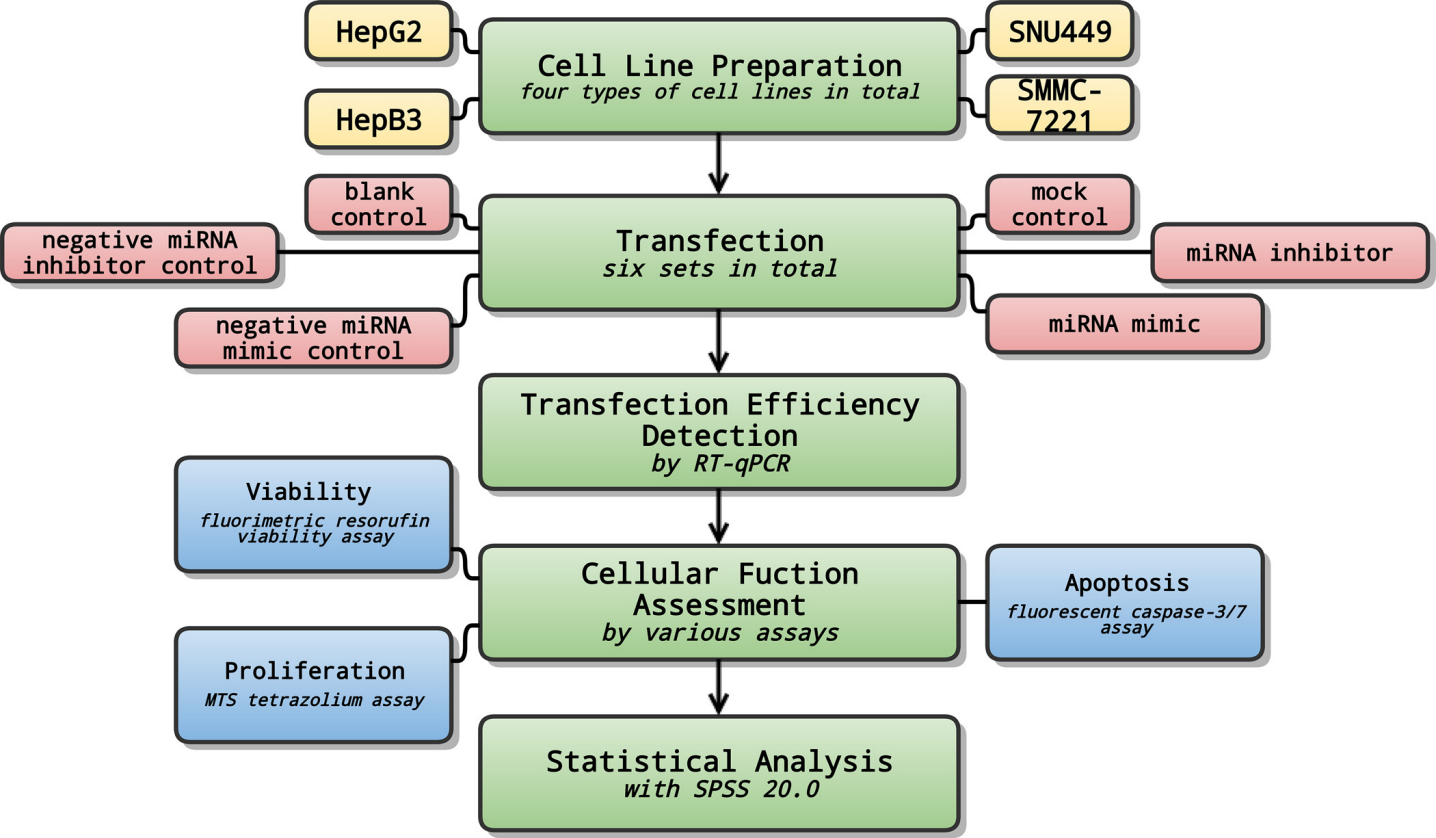
Flow chart of *in vitro* processes. *In vitro* experiments were performed to further verify the tumor-suppressive role of miR-132 and to assess its cellular functions in HCC.

**Fig 2:**
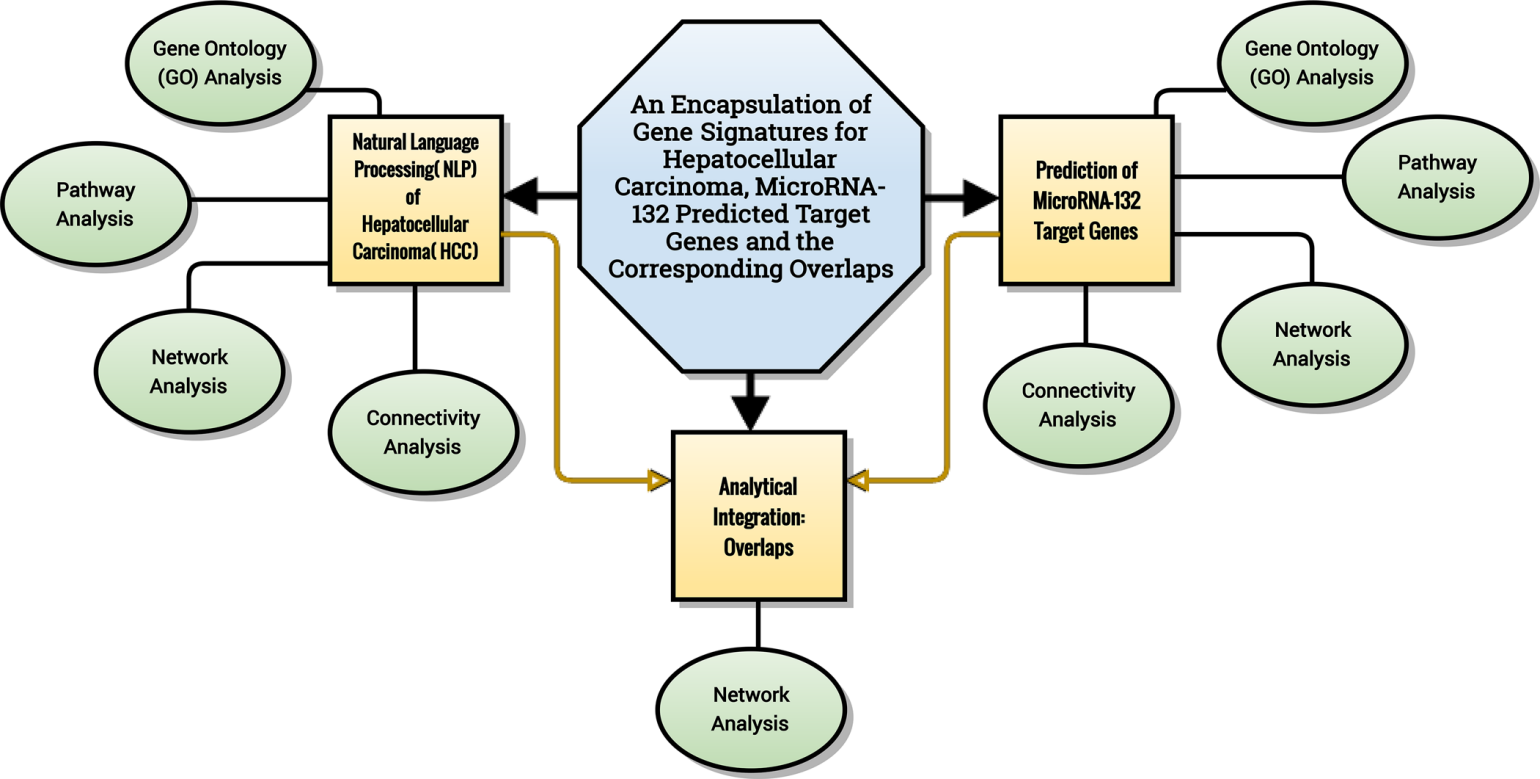
Flow chart of bioinformatic processes. A series of tasks, i.e. natural language processing (NLP) analysis of HCC, prediction of miRNA-132 target genes, comprehensive gene analyses and analytical integration was conducted successively.

*In vitro* experiments were performed to further verify the tumor-suppressive role of miR-132 and to assess its cellular functions in HCC (Fig 1). A series of tasks, i.e. natural language processing (NLP) analysis of HCC, prediction of miRNA-132 target genes, comprehensive gene analyses and analytical integration was then conducted successively (Fig 2).

### 2.1 Verification of role and assessment of cellular functions of miR-132 in HCC

#### 2.1.1 Cell line preparation

Four types of cell lines were cultured as formerly reported, i.e. HepG2 (American Type Culture Collection, ATCC), HepB3 (ATCC), SNU449 (ATCC) and SMMC-7221 (Chinese Academy of Medical Sciences) [16–19]. *In vitro* processes were conducted in triplicate. HCC cells were established in 96-well plates with 2.5 × 10^3^ cells per well and incubated at the temperature of 37 degree Celsius for 24 hours prior to transfection.

#### 2.1.2 Transfection

The transfection procedures were conducted respectively in cells of blank control, mock control, negative miRNA inhibitor control, miR-132 inhibitor, negative miRNA mimic control, and miR-132 mimic (Ambion, Life Technologies Grand Island, NY, USA) at the concentration of 200 nanomoles/L for up to 96 hours with combiMAGnetofection (OZBIOSCIENCES, Marseille Cedex 9, France) in accordance with manufacturers’ instructions. Sequence of miR-132 was uaacagucuacagccauggucg.

#### 2.1.3 Transfection efficiency detection

Meanwhile, RT-qPCR was employed to detect and monitor the transfection efficiency. The entire RNA with miRNA included was extracted from HCC cells in corresponding groups respectively. The housekeeping reference here was the combination of RNU6B and let-7a for the purpose of miR-132 expression detection. The primers for miR-132, RNU6B and let-7a were maintained in TaqMan MicroRNA Assays (4427975, Applied Biosystems, Life Technologies Grand Island, NY 14072 USA). Sequence of miRNA and references were: miR-132: 000457; RNU6B: 001093; let-7a: 000377. In terms of reverse transcription, a total volume of ten microliters was applied with TaqMan MicroRNA Reverse Transcription Kit (4366596, Applied Biosystems, Life Technologies Grand Island, NY 14072 USA) with the exact same reverse primers. Real-time qPCR was conducted with Applied Biosystems PCR7900 as previously described [16–19], which was in the charge of detecting and monitoring miRNA expression.

#### 2.1.4 Cellular function assessment

A series of assays, i.e. fluorimetric resorufin viability assay, MTS tetrazolium assay, and the fluorescent caspase-3/7 assay, was used, as formerly described[16–25], to access the cellular functions of HCC cell lines influenced by miR-132 mimics and miR-132 inhibitors, which were cellular viability, proliferation and apoptosis respectively.

#### 2.1.5 Statistical analysis

All the data were analyzed with SPSS 20.0 and presented in the way of means ± standard deviation (SD) from three independent, single experiments at least. One-way analysis of variance (ANOVA) test was applied for significance analysis of different groups. When statistical significance emerged in the probability for ANOVA, the least significant difference (LSD) method of multiple comparisons between two groups was employed. If the P value was less than 0.05, it would be regarded to be statistically significant.

### 2.2 NLP analysis of HCC

**Fig 3:**
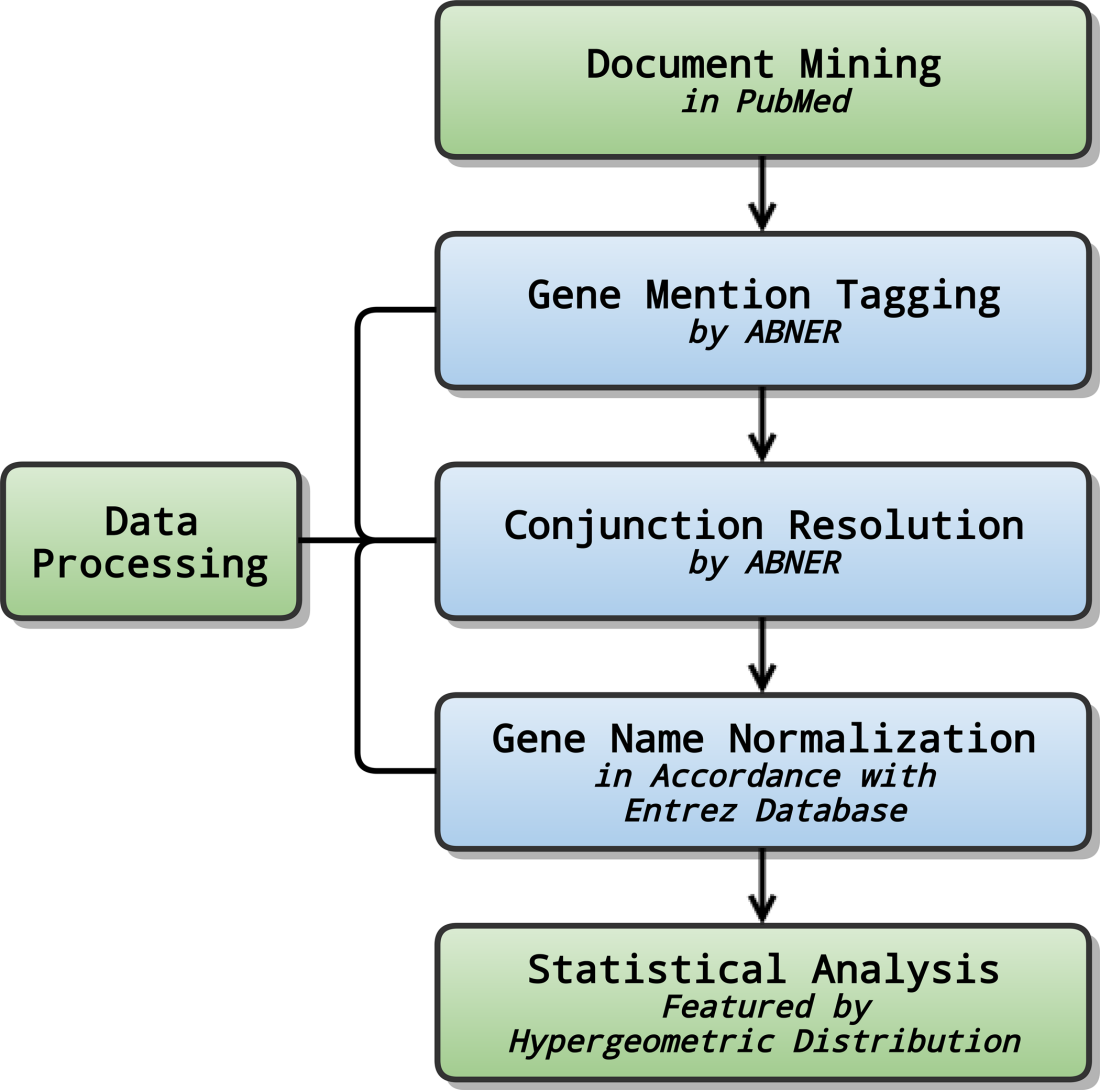
Flow chart of NLP analysis for HCC. The NLP analysis procedure of HCC includes document mining, data processing and statistical analysis.

The NLP analysis procedure of HCC was summarized in Fig 3.

#### 2.2.1 Document mining

An electronic search in PubMed was performed in an attempt to include all relevant articles published between January 1, 1980 and May 25, 2015. The combination of search strategy employed were as follows: (hepatocellular carcinoma) AND (resistance OR prognosis OR metastasis OR recurrence OR survival OR carcinogenesis OR sorafenib OR bevacizumab) and (‘1980/01/01” [PDAT]: “2015/05/25” [PDAT]). All the relevant proteins and genes related to the keywords above were mined out and a corresponding list was later created.

#### 2.2.2 Data processing

A Biomedical Named Entity Recognizer (ABNER), an open-source software (http://pages.cs.wisc.edu/~bsettles/abner/), was adopted for gene mention tagging, which automatically tagged out proteins, genes and other entity labels in the text. Conjunction resolution was also in the charge of ABNER, by which extracted genes were analytically resolved and sorted. For instance, ABNER would detect the “Caspase3/7” gene and resolve it into two separate genes, i.e. Caspase3 gene and Caspase7 gene. [26] Since multiple names might be used for the same gene, we normalized all the gene names in the articles into a standard set of names according to Entrez Database developed by NCBI.[27, 28]

#### 2.2.3 Statistical analysis

The frequency of occurrences was counted respectively for each gene. A higher frequency of a certain gene suggested a greater chance that the gene would be associated with HCC. The total number of articles in PubMed was labeled as N. The occurrence frequencies of genes and HCC in the PubMed were recorded as *m* and *n* respectively. *K* was set as the frequency of spontaneous co-occurrence of the specific gene and HCC under actual circumstances. With the assistance of hypergeometric distribution, it became possible to calculate the probability of occurrence frequency of co-citation greater than *k* under complete randomness by the formulae listed below:

$$p=1-\sum_{i=0}^{k-1}p(i|n,m,N)$$

$$p(i|n,m,N)=\frac{n!(N-n)!m!(N-m)!}{\left(n-i\right)!i!\left(n-m\right)!\left(N-n-m+i\right)!N!}$$

### 2.3 Prediction of miRNA-132 target genes

Eleven bioinformatics tools were employed to predict the potential target genes of miRNA-132, which are as follows: DIANA-microT[29], MicroInspector[30], miRanda[31], MirTarget2[32], miTarget[33], NBmiRTar[34], PicTar[35], PITA[36], RNA22[37], RNAhybrid[38] and TargetScan/TargetScanS[39]. Only when a predicted target gene were nominated by at least four bioinformatics tools would it be considered to be reliable and further eligible for its inclusion of the current study.

### 2.4 Comprehensive gene analyses

#### 2.4.1 Gene ontology (GO) analysis

It was the GSEABase package of the R Project for Statistical Computing (https://www.r-project.org/) that took on the responsibility for GO analysis, which categorized predicted target genes into groups according to three independent classification systems, i.e. molecular function, cellular component, and biological process.

#### 2.4.2 Pathway analysis

Potential target genes of miR-132 were mapped into the Kyoto Encyclopedia of Genes and Genomes (KEGG) Pathway Database with the assistance of GenMAPP v2.1[40], which was also employed for the calculation of enrichment *P* value of each pathway.

#### 2.4.3 Network analysis

In general, three different classes of interaction relationships were integrated in attendance with the following principles:

- Existing data from protein interaction, gene regulation and protein modification mentioned in KEGG Database.
- Available data from high-throughput experiments. A case in point was a yeast-two-hybrid system which supported the protein-protein interaction.
- Published or reported data with regard to interactions among genes.

The specific steps could be described as follows:

- For data mentioned in KEGG Database, pathway data downloaded from KEGG Pathway Database were ported into the R Project for Statistical Computing (https://www.r-project.org/) and the KEGGSOAP package (http://www.bioconductor.org/packages/2.4/bioc/html/KEGGSOAP.html) was employed for the performance of a genome-wide interaction analysis, which included three types of relationships listed in Table 1.
- For data from high-throughput experiments, data regarding protein-protein interactions were obtained from the MIPS Mammalian Protein-Protein Interaction Database (http://mips.helmholtz-muenchen.de/proj/ppi/).
- For the published or reported data, they proceeded with the algorithm described in Section 2.1.3.

**Table 1:** Three classes of relationships are mentioned in the genome-wide interaction analysis.

ECrel	enzyme-enzyme relation, indicating two enzymes catalyzing successive reaction steps
PPrel	protein-protein interaction, such as binding and modification
GErel	gene expression interaction, indicating relation of transcription factor and target gene product

At length, we took the all the above factors and data into account comprehensively, established the corresponding networks and displayed then via figures with the assistance of MEDUSA software.

#### 2.4.4 Connectivity analysis

Connectivity analysis was used for the display of degrees to which genes/proteins interacts with one another.

### 2.5 Analytical integration

The gene overlaps were integrated analytically between HCC-related genes from NLP analysis and predicted target genes of miR-132 by bioinformatics softwares.

## 3. Results

### 3.1 The role and cellular functions of miR-132 in HCC

RT-qPCR was employed to detect and monitor the influences of different agents on the expression levels of miR-13, which guaranteed the transfection efficiency to be optimal. The impact of miR-132 on cell growth was established with two different methods respectively, i.e. fluorimetric resorufin viability assay and MTS tetrazolium assay.

**Fig 4:**
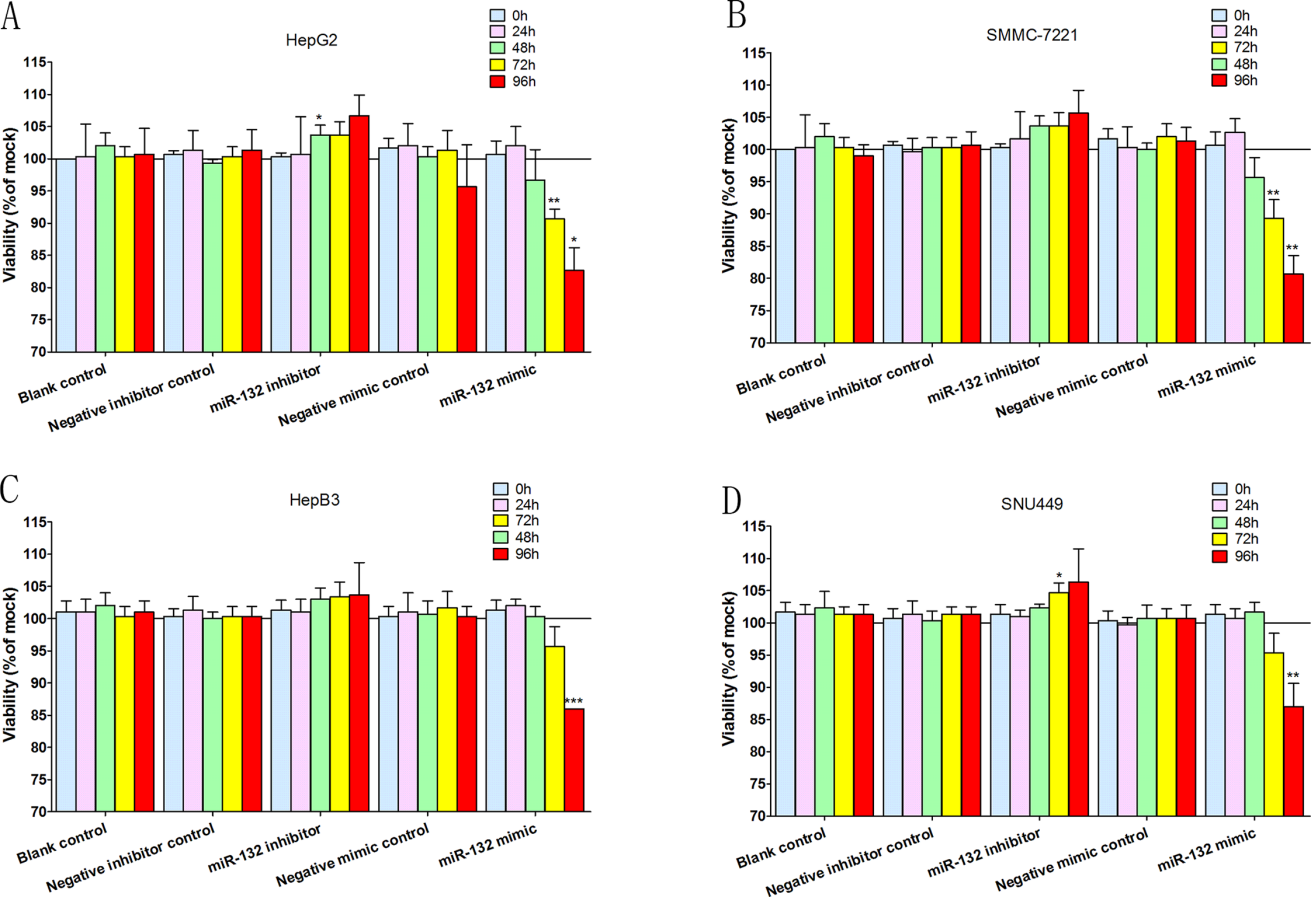
Viability test. Time-dependent effects of miR-132 were assessed on viability in various HCC cell lines, i.e. HepG2 (A), SMMC-7221 (B), HepB3 (C) and SNU449 (D). Columns represent the averages of sets of three single, independent experiments while bars stand for the standard deviations. *P < 0.05, ** P < 0.01 and ***P < 0.001, compared to blank and negative controls at the same time point.

In the fluorimetric resorufin viability assay (Fig 4), both the miR-132 mimic and the miR-132 inhibitor had certain influence on the viability of cells. After the transfection with miR-132 mimic, cell viability significantly decreased in all the four cell lines: both HepG2 and SMMC-7221cell lines showed the similar tendencies, i.e. the viability both declined to around 90% and approximately 80% after 72 hours and 96 hours of the transfections of the mimics; as for HepB3 and SNU449, the viability of both cell lines dropped to around 85% after 96 hours of the transfections of the mimics. However, the less significant results emerged when miR-132 inhibitors was transfected: only after 48 hours and 72 hours of inhibitor transfection into HepG2 and SNU449 cell lines respectively, did a slight increase of less than 5% appear in both cell lines; SMMC-7221 and HepB3 cell lines displayed no results of statistical significance after being transfected with miR-132 inhibitors.

**Fig 5:**
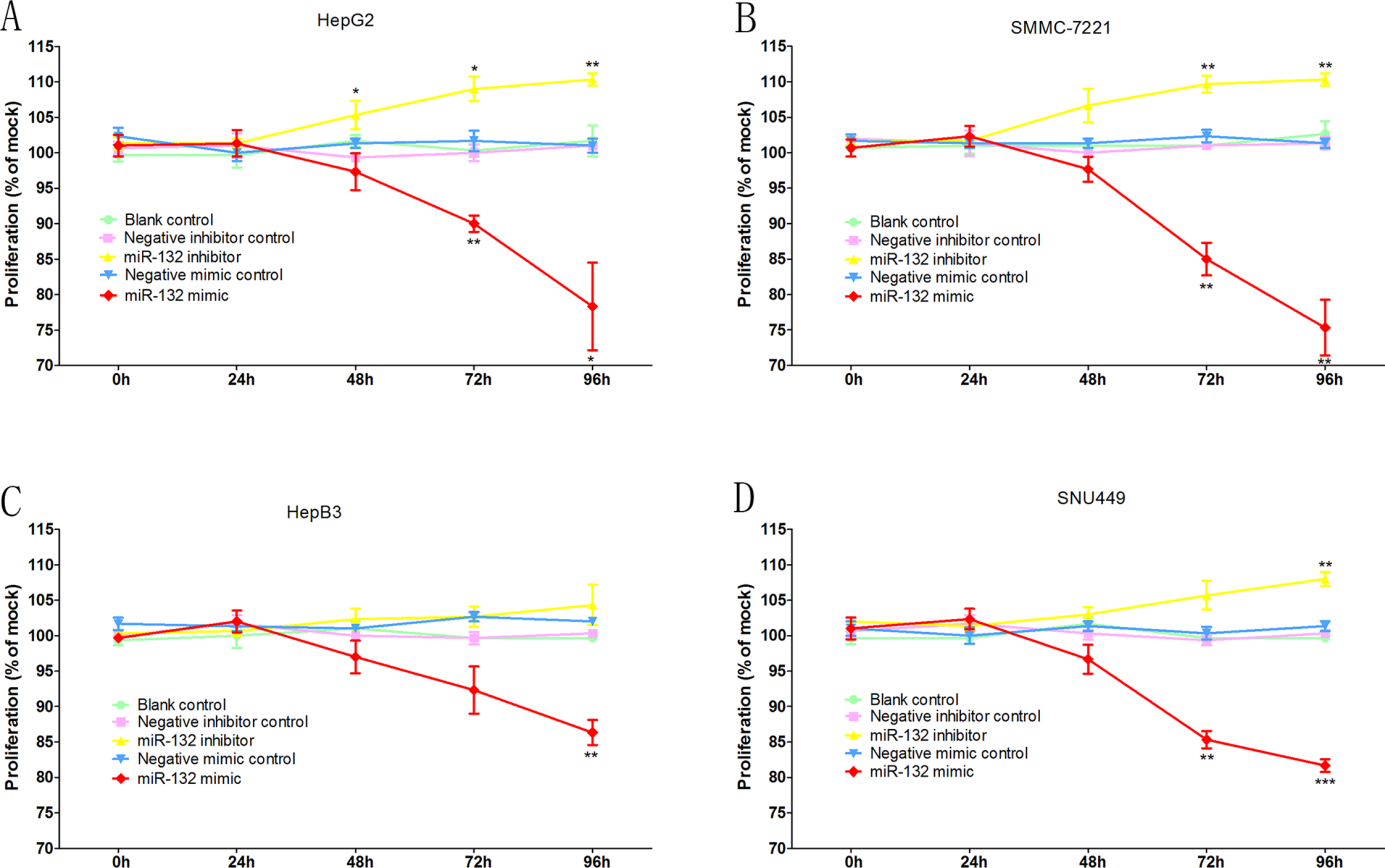
Proliferation test. Time-dependent effects of miR-132 were assessed on proliferation in various HCC cell lines, i.e. HepG2 (A), SMMC-7221 (B), HepB3 (C) and SNU449 (D). Points represent the averages of sets of three single, independent experiments while bars stand for the standard deviations. *P < 0.05, ** P < 0.01 and ***P < 0.001, compared to blank and negative controls at the same time point.

The cell proliferation was assessed by the MTS tetrazolium assay (Fig 5), in which the observation resembled the result in the fluorimetric resorufin viability assay—miR-132 mimics showed greater impacts on cell lines than miR-132 inhibitors did. After being transfected with miR-132 mimics, the proliferation in all the four cell lines dropped significantly: HepG2 cell line showed a post-72hrs decline of approximately 10% and a post-96hrs drop of around 20% in proliferation; SMMC-7721 cell line displayed a post-72hrs decrease of approximately 15% and a post-96hrs fall of around 25%; a post-96hrs downward trend of approximately 15% was observed in HepB3 cell line; and SNU499 cell line demonstrated a post-72hrs reduce of around 15% and a post-92hrs decline of approximately 20%. MiR-132 inhibitors displayed less significant tendencies than miR-132 mimics did in terms of proliferation as well: the proliferation rate rose by an approximate 5% to 10% after the 48–72 hours of inhibitor transfection in HepG2 cell line; a post-72hrs increase of approximately 10% was observed in SMMC-7721 cell line posterior to the transfection and the trend remained roughly stable till the post-96hrs detection; SNU449 cell line showed an approximate uptrend of 7% only after 96 hours of the transfection; and no statistically significant data of proliferation was observed in HepB3 posterior to the inhibitor transfection.

**Fig 6:**
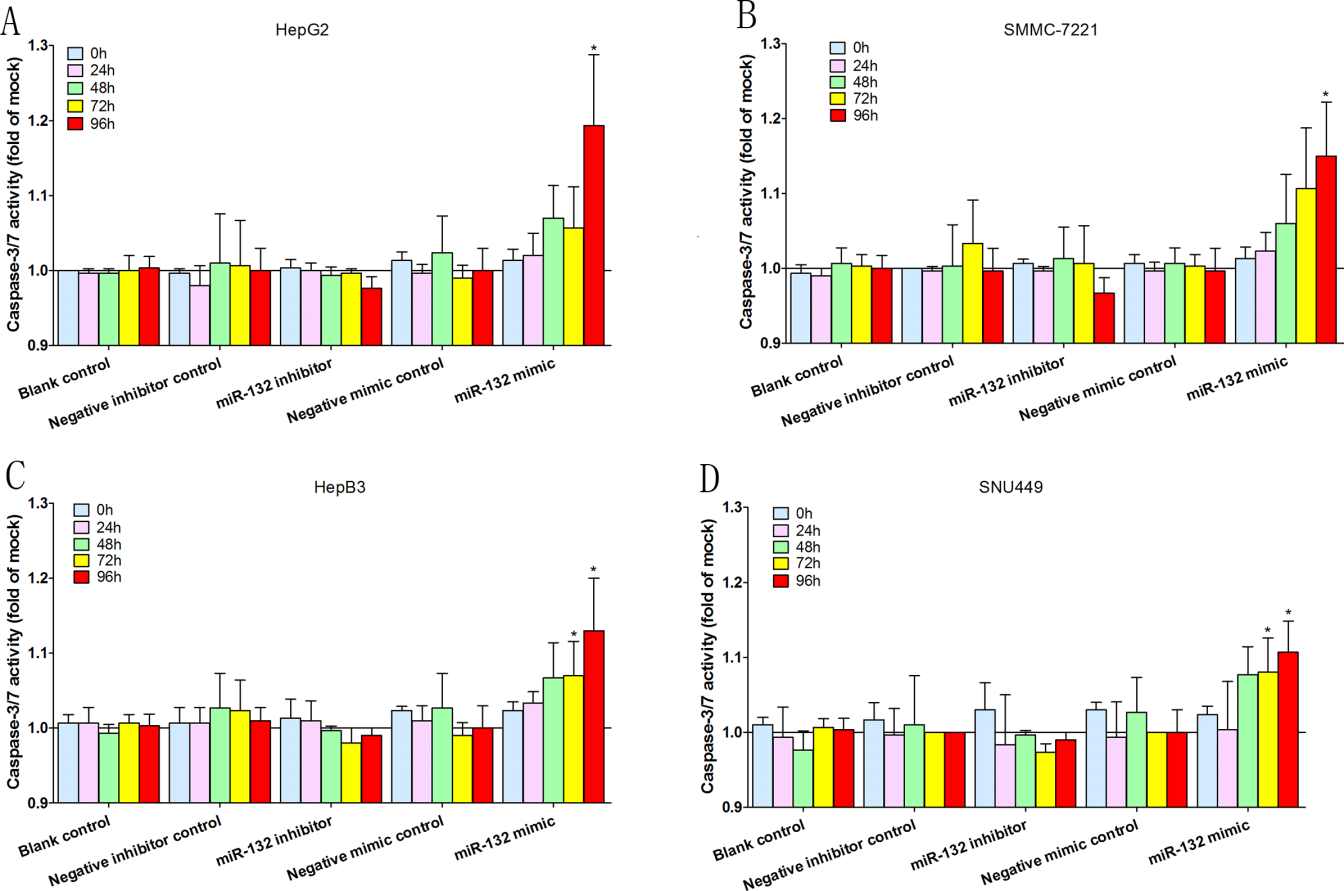
Apoptosis test. Time-dependent effects of miR-132 were assessed on the caspase-3/7 activities in various HCC cell lines, i.e. HepG2 (A), SMMC-7221 (B), HepB3 (C) and SNU449 (D). Points represent the averages of sets of three single, independent experiments while bars stand for the standard deviations. *P < 0.05, compared to blank and negative controls at the same time point.

The fluorescent caspase-3/7 assay was performed to investigate the effects of miR-132 on the apoptosis and caspase activation in HCC cells (Fig 6). Similar to the viability and proliferation tests, miR-132 mimics were much more influential than miR-132 inhibitors in this part. After the transfection with miR-132 mimics, the caspase-3/7 activity was significantly upturned by a post-96hrs fold change of approximately 0.2 in both HepG2 and SMMC-7221 cell lines. More significant results emerged in both HepB3 and SNU499 cell lines, where we observed a statistically significant, constant yet slight increase of the caspase-3/7 activity after both 72 hours and 92 hours of the mimics’ transfection. Opposite to the mimics’ performance, miR-132 inhibitors demonstrated no statistically significant impact on the apoptosis and caspase activation in HCC cells.

### 3.2 NLP analysis of HCC

**Fig 7:**
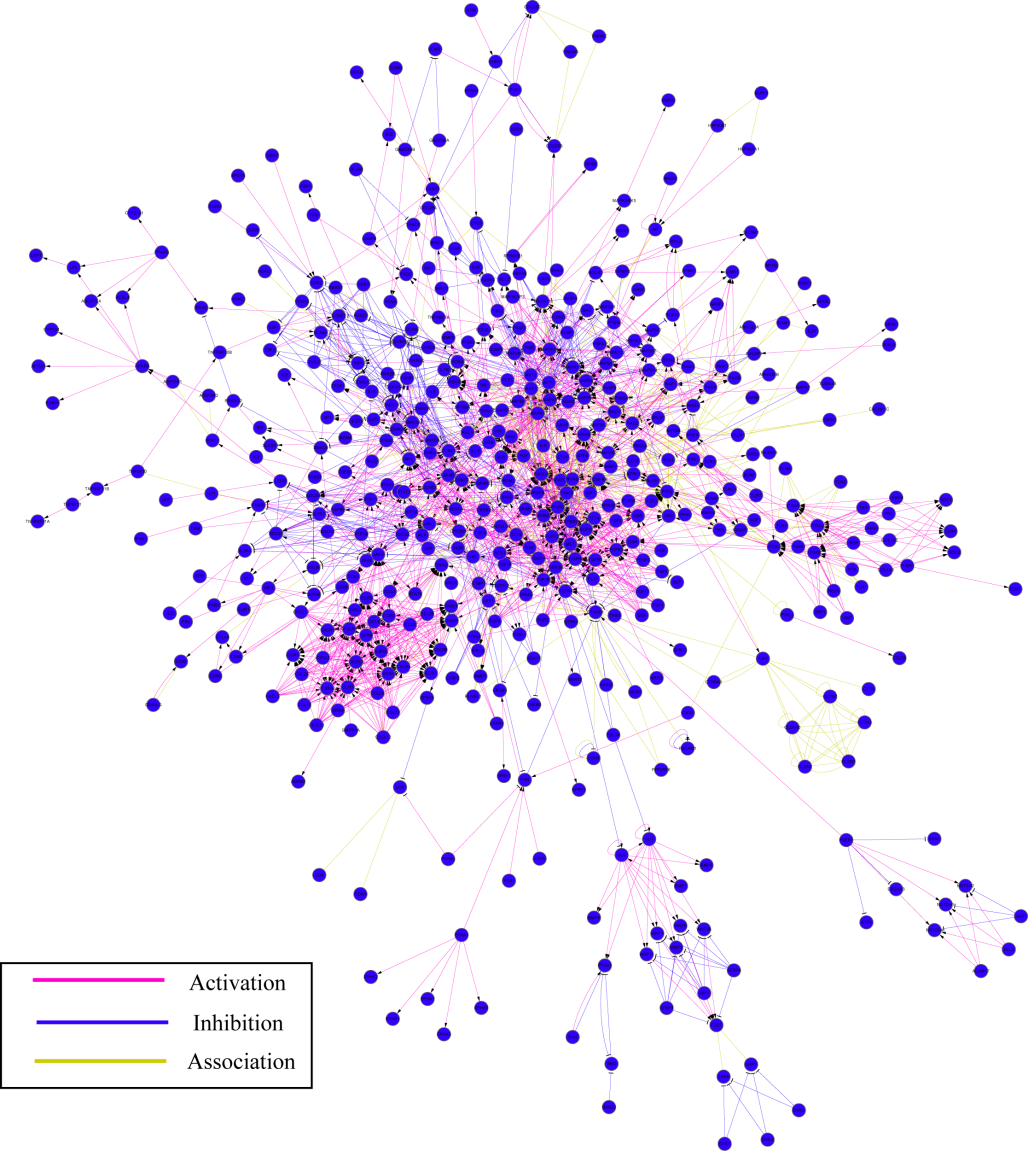
Network analysis for HCC. In NLP analysis, a network of multiple genes was established for HCC.

**Fig 8:**
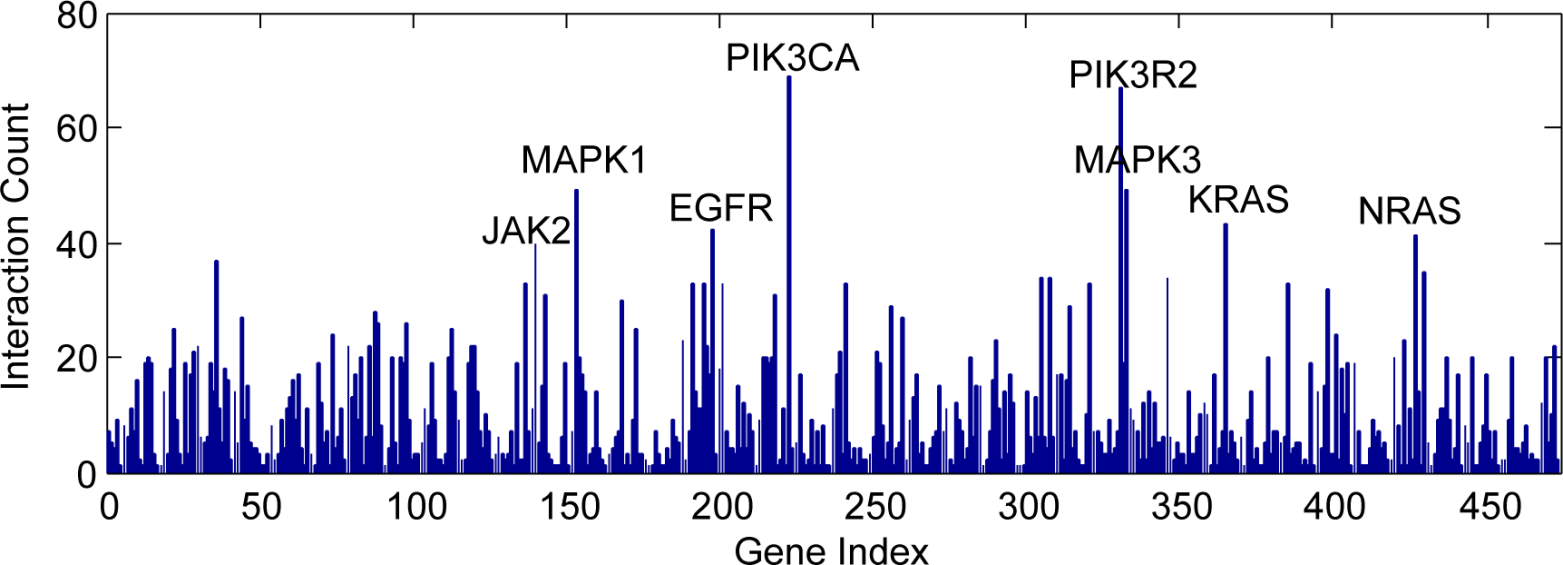
Connectivity analysis for HCC. Connectivity analysis demonstrated that the top connectivities of PIK3CA and PIK3R2.

A total of 64,577 records of titles and abstracts related to HCC were identified in PubMed, with 1800 related genes obtained. Subsequently, GO analysis, pathway analysis and network analysis were further conducted. Firstly, GO analysis classified all the obtained genes in accordance with molecular function, cellular component and biological process, which were summarized in Table 2. Later, 24 pathways were proved significant (P< = 0.05) by pathway analysis (Table 3). Lastly, network analysis was carried out in order to elucidate how genes could possibly interact with or regulate each other. Hub genes could be defined as the highly connected genes in the network, which were considered vital in gene regulation and thus affect the stability of the network. In the current study, a network of genes was established and shown in Fig 7. In addition, connectivity analysis was employed, highlighting the top connectivity of PIK3CA and the second highest connectivity of PIK3R2 (Fig 8).

**Table 2:** GO analysis classified all the HCC-related genes obtained from NLP in accordance with molecular function, cellular component and biological process.

Molecular function		
Term	count	P-value
transcription regulatory activity	194	1.27E-10
transporter activity	69	0.999018385
signal transduction activity	384	0.000166595
enzyme regulator activity	84	0.000951267
Translation activity	4	0.647613656
Nucleic acid binding activity	327	2.22E-10
extracellular structural activity	3	0.416191131
kinase activity	173	1.04E-10
cytoskeletal activity	51	0.256119892
Other molecular function	1290	1.73E-10
Cellular component		
Term	count	P-value
extracellular matrix	56	5.61E-08
non-structural extracellular	250	7.83E-11
Cytosol	65	6.08E-08
plasma membrane	345	1.43E-10
Other membranes	603	0.999999471
Nucleus	536	1.48E-10
Cytoskeleton	108	0.018083091
translational apparatus	17	0.704983031
Mitochondrion	82	0.846509924
ER/Golgi	117	0.542990563
Other cytoplasmic organelle	47	0.281825841
Other cellular component	774	1.57E-10
Biological process		
Term	count	P-value
cell cycle and proliferation	350	1.13E-10
stress response	235	1.02E-10
Transport	242	0.15918636
developmental processes	531	1.23E-10
RNA metabolism	339	5.22E-09
DNA metabolism	89	2.41E-11
protein metabolism	434	1.40E-10
Other metabolic processes	370	1.39E-10
cell organization and biogenesis	305	1.24E-10
cell-cell signaling	67	2.67E-09
signal transduction	453	4.06E-10
cell adhesion	107	5.35E-10
Death	221	1.17E-10
Other biological processes	705	0.000230959

**Table 3:** Twenty-four pathways were identified to be statistically significant for the NLP analysis of HCC (P< = 0.05).

Pathway	Count	P-value
Cytokine-cytokine receptor interaction	111	1.41E-17
Focal adhesion	85	1.37E-12
Neurotrophin signaling pathway	61	6.97E-12
Toll-like receptor signaling pathway	53	1.91E-11
MAPK signaling pathway	100	3.92E-11
p53 signaling pathway	39	2.62E-09
Chemokine signaling pathway	74	6.69E-09
Cell cycle	56	1.28E-08
Apoptosis	44	2.29E-08
ErbB signaling pathway	44	2.29E-08
T cell receptor signaling pathway	50	4.63E-08
Natural killer cell mediated cytotoxicity	57	7.02E-08
Adherens junction	38	1.67E-06
Jak-STAT signaling pathway	59	7.41E-06
Fc epsilon RI signaling pathway	35	1.64E-04
NOD-like receptor signaling pathway	30	2.18E-04
Wnt signaling pathway	53	0.001007886
TGF-beta signaling pathway	36	0.001133761
Cell adhesion molecules (CAMs)	48	0.001159562
VEGF signaling pathway	32	0.002400693
Adipocytokine signaling pathway	29	0.00582618
Insulin signaling pathway	47	0.006083566
B cell receptor signaling pathway	31	0.007834751
Hematopoietic cell lineage	32	0.043504802

### 3.3 The analysis of miR-132 predicted target genes

Eleven bioinformatics softwares aforementioned were used for the prediction of potential target genes of miRNA-132 and we would only include a certain target gene if it got nominated by at least four bioinformatics softwares. As a result, 501 potential target genes were considered qualified and later went through GO analysis, pathway analysis and network analysis.

**Fig 9:**
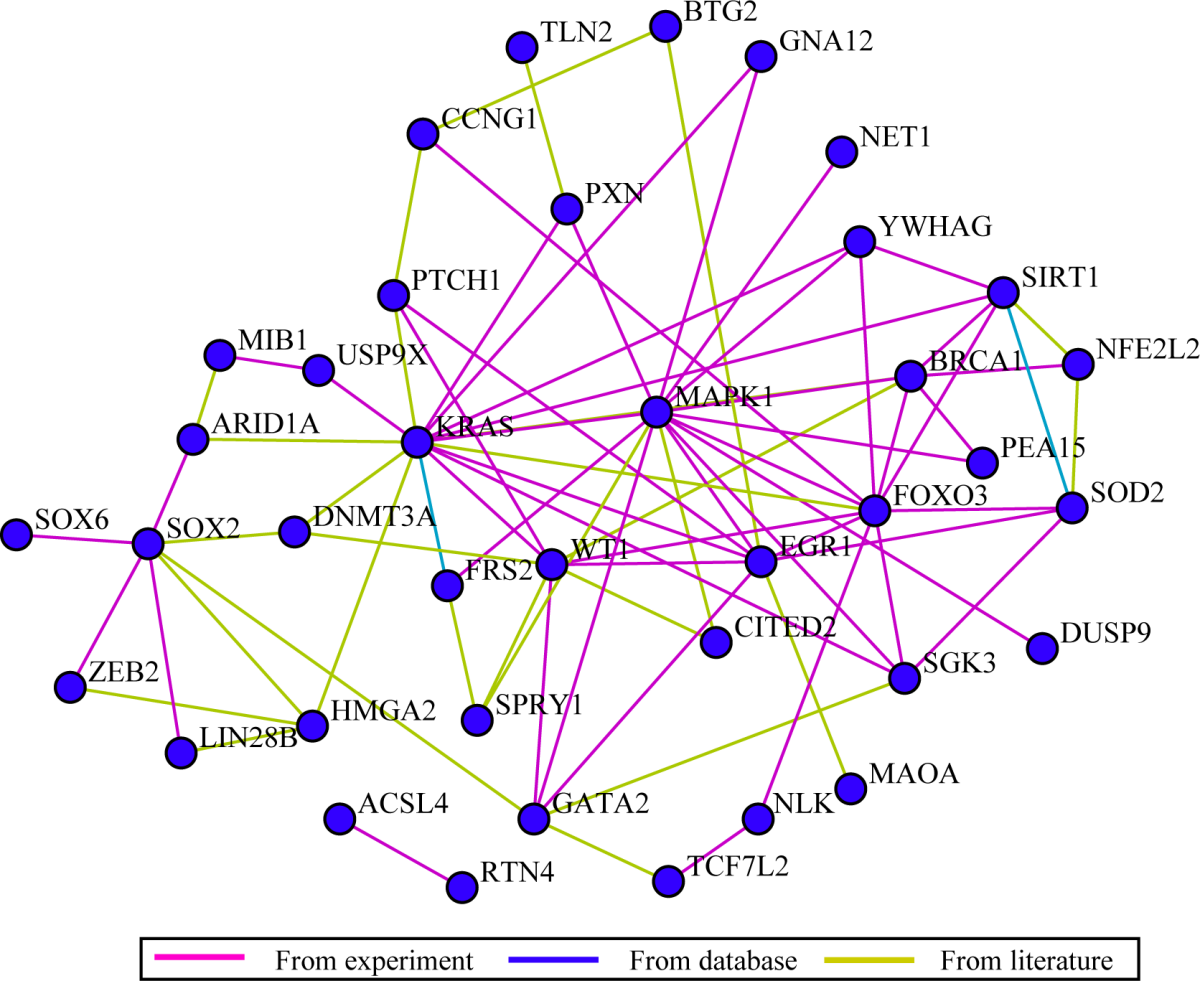
Network analysis for miR-132 predicted target genes. Network analysis provided insights into the potential interacting and regulatory networks of miR-132.

**Fig 10:**
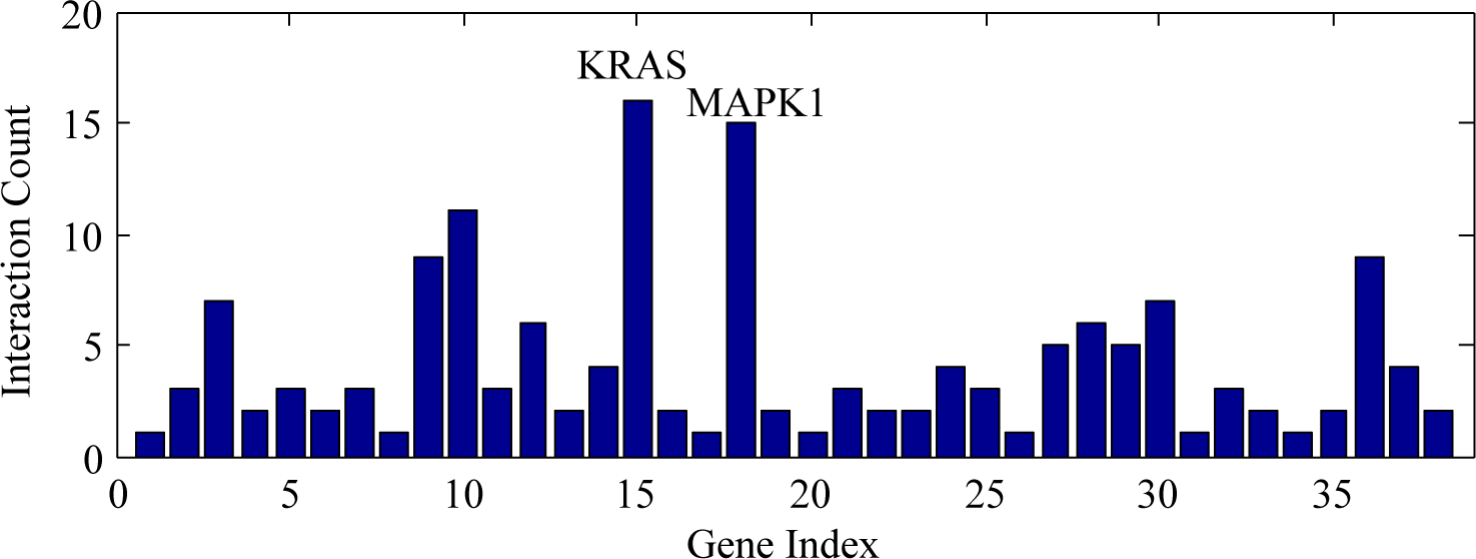
Connectivity analysis for miR-132 predicted target genes. The additional connectivity analysis revealed that KRAS harbored the highest connectivity and MAPK1 the second highest, interacting with sixteen and fifteen genes respectively.

To begin with, all the miR-132 predicted target genes were sorted out according to molecular function, cellular component and biological process by GO analysis (Table 4). Furthermore, pathway analysis identified a total of 70 pathways (S1 Table) and four of them were considered statistically significant (P< = 0.05), i.e. neurotrophin signaling pathway (count = 5; P = 0.002082; MAPK1, YWHAG, KRAS, FOXO3, FRS2), MAPK signaling pathway (count = 5; P = 0.029881; MAPK1, KRAS, NLK, GNA12, DUSP9), VEGF signaling pathway (count = 3; P = 0.044476; MAPK1, KRAS, PXN), and adherens junction (count = 3; P = 0.046643; MAPK1, NLK, TCF7L2). Network analysis provided us with an unprecedentedly clear map on the potential interacting and regulatory networks of miR-132 (Fig 9). The additional connectivity analysis revealed that KRAS harbored the highest connectivity among all the hub genes in the network of miR-132 predicted genes, interacting with sixteen genes (ARID1A, BRCA1, DNMT3A, EGR1, FOXO3, FRS2, GNA12, HMGA2, MAPK1, PTCH1, PXN, SGK3, SIRT1, USP9X, WT1, YWHAG) (z-test, P = 0.00618). Besides, MAP1 ranked as the second highest connected hub genes among all, with fifteen genes interacted (BRCA1, CITED2, DUSP9, EGR1, FOXO3, FRS2, GATA2, GNA12, KRAS, NET1, PEA15, PXN, SGK3, SPRY1, and YWHAG) (z-test, P = 0.001543). (Fig 10, S2 Table)

**Table 4:** All the miR-132 predicted target genes were sorted out according to molecular function, cellular component and biological process by GO analysis.

Molecular function		
Term	count	P-value
transcription regulatory activity	15	1.16E-06
transporter activity	2	0.854486
signal transduction activity	5	0.994435
enzyme regulator activity	1	0.88333
nucleic acid binding activity	21	2.25E-05
kinase activity	6	0.05029
cytoskeletal activity	2	0.485943
other molecular function	48	0.112394
Cellular component		
Term	Count	P-value
mitochondrion	3	0.648952
other cytoplasmic organelle	3	0.20695
Cytosol	3	0.11775
cytoskeleton	4	0.408324
Nucleus	30	5.98E-06
plasma membrane	9	0.569293
other membranes	18	0.983114
translational apparatus	1	0.499061
ER/Golgi	4	0.631317
other cellular component	33	0.305633
Biological process		
Term	count	P-value
cell cycle and proliferation	12	0.001377
Transport	6	0.882846
stress response	7	0.114018
developmental processes	28	1.13E-08
RNA metabolism	23	2.57E-05
DNA metabolism	4	0.060241
other metabolic processes	12	0.310527
cell organization and biogenesis	15	0.002505
cell-cell signaling	5	0.00704
signal transduction	18	0.101181
cell adhesion	3	0.38637
protein metabolism	17	0.013399
Death	9	0.002664
other biological processes	25	0.474989

### 3.4 Analytical integration of results from NLP procedure of HCC and prediction of miRNA-132 target genes

**Fig 11:**
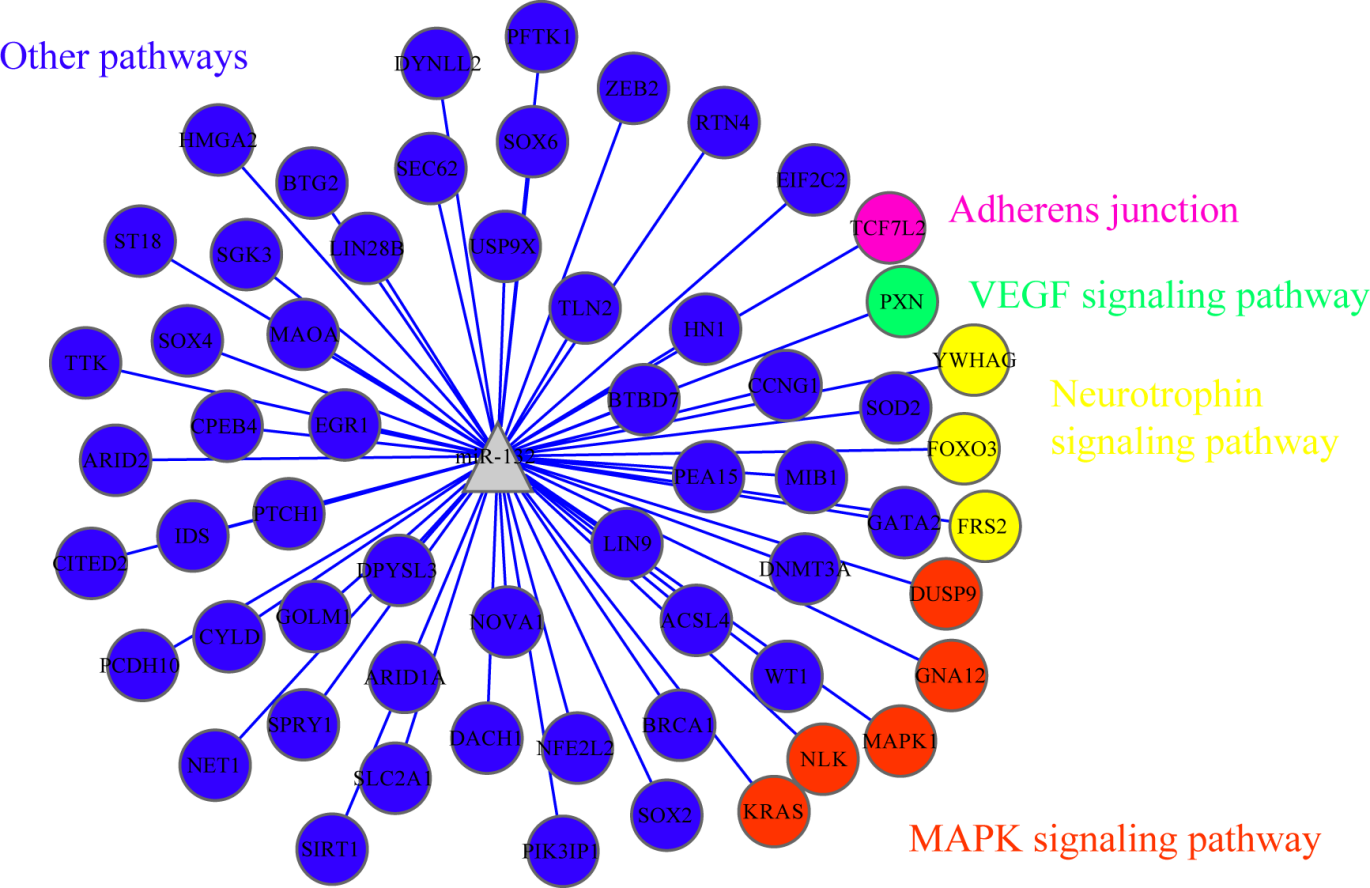
Network analysis for the overlapped genes in the analytical integration. A network analysis was performed among the fifty-nine genes identified in the analytical integration so as to better comprehend the possible underlying mechanisms.

The integration systematically analyzed the overlaps and featured fifty-nine genes that were both potentially HCC-related and probably regulated by miR-132 (Table 5). A network analysis was performed among the fifty-nine genes identified (Fig 11) so as to better comprehend the possible underlying mechanisms. MiR-132 might be associated with TCF7L2 via adherens junction and interact with PXN via VEGF signaling pathway. Neurotrophin signaling pathway might mediate the interactions and regulations between miR-132 and YWHAG, FOXO3 and FRS2. MAPK signaling pathway could be responsible for the communications between miR-132 and DUSP9, GNA12, MAPK1, NLK and KRAS. The remaining seventy-two genes would interact with miR-132 via other different pathways.

**Table 5:** The integration systematically analyzed the overlaps and featured 59 genes that were both potentially HCC-related and probably regulated by miR-132.

Gene	P-value	Gene Description
SIRT1	<1.00E-08	sirtuin (silent mating type information regulation 2 homolog) 1 (S. cerevisiae)
SPRY1	0.000332	sprouty homolog 1, antagonist of FGF signaling (Drosophila)
DPYSL3	0.000387	dihydropyrimidinase-like 3
NOVA1	0.028707	neuro-oncological ventral antigen 1
SOX4	2.83E-05	SRY (sex determining region Y)-box 4
PFTK1	0.024657	PFTAIRE protein kinase 1
SEC62	0.01855	SEC62 homolog (S. cerevisiae)
MAPK1	0.000255	mitogen-activated protein kinase 1
PXN	9.25E-06	Paxillin
PCDH10	0.022625	protocadherin 10
BTG2	0.003519	BTG family, member 2
HMGA2	2.47E-05	high mobility group AT-hook 2
YWHAG	0.10812	tyrosine 3-monooxygenase/tryptophan 5-monooxygenase activation protein, gamma polypeptide
PEA15	0.002455	phosphoprotein enriched in astrocytes 15
HN1	0.01855	hematological and neurological expressed 1
SGK3	0.05855	serum/glucocorticoid regulated kinase family, member 3
LIN28B	1.29E-05	lin-28 homolog B (C. elegans)
SOX6	0.03475	SRY (sex determining region Y)-box 6
ARID2	0.000332	AT rich interactive domain 2 (ARID, RFX-like)
ZEB2	<1.00E-08	zinc finger E-box binding homeobox 2
DUSP9	0.010348	dual specificity phosphatase 9
SOX2	<1.00E-08	SRY (sex determining region Y)-box 2
FOXO3	1.62E-08	forkhead box O3
TLN2	0.03274	talin 2
CPEB4	0.008287	cytoplasmic polyadenylation element binding protein 4
TCF7L2	0.008234	transcription factor 7-like 2 (T-cell specific, HMG-box)
DNMT3A	1.98E-06	DNA (cytosine-5-)-methyltransferase 3 alpha
MIB1	<1.00E-08	Mindbomb homolog 1 (Drosophila)
WT1	0.069664	Wilms tumor 1
LIN9	0.02059	lin-9 homolog (C. elegans)
CCNG1	0.0008	cyclin G1
KRAS	0.005874	v-Ki-ras2 Kirsten rat sarcoma viral oncogene homolog
PIK3IP1	0.022625	phosphoinositide-3-kinase interacting protein 1
GATA2	0.093154	GATA binding protein 2
ARID1A	9.06E-07	AT rich interactive domain 1A (SWI-like)
DYNLL2	0.000192	dynein, light chain, LC8-type 2
EGR1	0.041332	early growth response 1
TTK	0.05855	TTK protein kinase
IDS	0.13732	iduronate 2-sulfatase
MAOA	0.40307	monoamine oxidase A
BTBD7	4.30E-05	BTB (POZ) domain containing 7
PTCH1	2.86E-05	patched homolog 1 (Drosophila)
USP9X	0.044739	ubiquitin specific peptidase 9, X-linked
CYLD	<1.00E-08	cylindromatosis (turban tumor syndrome)
SOD2	0.004207	Superoxide dismutase 2, mitochondrial
ST18	0.014458	suppression of tumorigenicity 18 (breast carcinoma) (zinc finger protein)
EIF2C2	1.12E-06	Eukaryotic translation initiation factor 2C, 2
BRCA1	0.65001	breast cancer 1, early onset
GNA12	4.29E-06	guanine nucleotide binding protein (G protein) alpha 12
NLK	0.040756	nemo-like kinase
GOLM1	<1.00E-08	golgi membrane protein 1
DACH1	0.046724	Dachshund homolog 1 (Drosophila)
ACSL4	1.15E-05	acyl-CoA synthetase long-chain family member 4
FRS2	0.074089	fibroblast growth factor receptor substrate 2
RTN4	0.13373	reticulon 4
SLC2A1	0.003185	solute carrier family 2 (facilitated glucose transporter), member 1
NET1	0.036756	neuroepithelial cell transforming 1
CITED2	0.040756	Cbp/p300-interacting transactivator, with Glu/Asp-rich carboxy-terminal domain, 2
NFE2L2	0.002581	nuclear factor (erythroid-derived 2)-like 2

## 4. Discussion

Since HCC ranks top as one of the most frequent cancers internationally, novel biomarkers for early diagnosis and precise prognosis of HCC are pressingly demanded [1]. Thus, researchers’ attention has been drawn by microRNAs, a diverse class of small non-coding RNAs, which regulate and mediate gene expressions in human oncogenesis [5, 6]. Among them is the vigorously studied miR-132, which has displayed its associations with several cancers including colorectal cancer[8], gastric cancer[9], and pancreatic cancer[12].

Originally, Wei, et al. [14] reported the potentially active and crucial role of miR-132 in HBV-mediated hepatocarcinogenesis, and the down-regulation of miR-132 in HBV-related HCC with a sample size of 20 patients. To complement their research, our previous study employed an almost quintupled cohort of 95 patients to verify the decrease of miR-132 in HCC and investigated the relationships between miR-132 and mainstream clinical/pathological parameters as well as recurrence data in HCC patients, confirming the tumor-suppressive role of miR-132 in HCC *(Xin Zhang*, *et al*. *Down-regulation of MicroRNA-132 Indicates Progression in Hepatocellular Carcinoma*: *A Clinical Perspective*. *Experimental and Therapeutic Medicine*. *In Press*.*)*. The associations have been proved statistically significant between the expression of miR-132 and metastasis, clinical TNM stage, and tumor capsular infiltration *(Xin Zhang*, *et al*. *Down-regulation of MicroRNA-132 Indicates Progression in Hepatocellular Carcinoma*: *A Clinical Perspective*. *Experimental and Therapeutic Medicine*. *In Press*.*)*. It was also speculated that its tumor-suppressive function in HCC would probably rely on suppressing CCNE1 expression[11], targeting ZEB2[8], HN1[7], Sox5[41], or ZEB2[42], provoking acetylcholinesterase-independent apoptosis[43], or mediating methylation-silencing and anti-metastasis in the controlling cellular adhesion of prostate cancer [13], which were established by researches on molecular mechanisms of miR-132 as a tumor suppressor in other malignancies. Furthermore, a study led by Liu, et al[15] identified the downregulation of miR‑132 in HCC tissues and cell lines. And its expression negatively correlated with tumor differentiation, TNM stage and lymph node metastasis. Also, miR-132 is believed to suppress cell proliferation, colony formation, migration and invasion, and induce apoptosis in HCC cells via *in vitro* experiments. *In vivo* demonstrated the role of miR-132 as a suppressor for tumor growth in nude mouse model. Lastly, they confirmed that PIK3R3 is a novel target gene of miR‑132 and miR‑132 might exert its tumor suppressive function by directly targeting the PIK3R3 and regulating the AKT/mTOR pathway.

However, no well-founded insights with supporting data were provided into the overview of potential molecular mechanisms of miR-132 in HCC [14][15] *(Xin Zhang*, *et al*. *Down-regulation of MicroRNA-132 Indicates Progression in Hepatocellular Carcinoma*: *A Clinical Perspective*. *Experimental and Therapeutic Medicine*. *In Press*.*)*, which calls for studies like the current one from us. Thanks to the booming bioinformatics technology, such as natural language processing, prediction of target genes, gene signatures and so forth, it is now possible for us to illuminate the potential targets, pathways or even regulatory networks of miR-132 in HCC [4, 26–39].

In the current study, *in vitro* experiments have been done to validated the tumor-suppressive role and assess the cellular functions of miR-132 in HCC and a series of tasks has been completed, including data mining and selecting, target genes prediction, comprehensive analyses (gene ontology analysis, pathway analysis, network analysis and connectivity analysis), and analytic integration, in order to elucidate HCC-related and miR-132-related potential targets, pathways and networks as well as those of the overlapped section.

The influences of miR-132 on various cellular functions in HCC were evaluated with different assays. Generally, both mimics and inhibitors of miR-132 were found to be able to influence cell functions of HCC cell lines though miR-132 mimics exerted greater influence on HCC cell lines than miR-132 inhibitors did. In viability test, cell viability significantly decreased in all the four cell lines transfected with miR-132 mimics while miR-132 inhibitors only increased the viability of two cell lines (HepG2 and SNU449) out of four (Fig 4). Observations from the proliferation test resembled those from the viability test. MiR-132 mimics made the proliferation rate significantly drop in all the four cell lines while its inhibitors displayed boosting role in the proliferation of three cell lines (HepG2, SMMC-7721, and SNU449) (Fig 5). In the apoptosis assessment, miR-132 mimics were considered effective in significantly upturning the caspase-3/7 activity in all the four cell lines (Fig 6), which means that miR-132 mimics positively affected the apoptosis process of HCC cells. However, no statistically significant observations were made with regards to the role of miR-132 inhibitors in the apoptosis and caspase activation in HCC cells (Fig 6). The above demonstrated that miR-132 is effective in both impeding HCC cell growth (decreasing viability and undermining proliferation) and boosting HCC cell apoptosis (upturning the caspase-3/7 activity), which mutually supports the findings by both Wei, et al. [14], Liu, et al.[15] and us *(Xin Zhang*, *et al*. *Down-regulation of MicroRNA-132 Indicates Progression in Hepatocellular Carcinoma*: *A Clinical Perspective*. *Experimental and Therapeutic Medicine*. *In Press*.*)*. Taking all the *in vitro* experiments and clinical expression data [14] [15] *(Xin Zhang*, *et al*. *Down-regulation of MicroRNA-132 Indicates Progression in Hepatocellular Carcinoma*: *A Clinical Perspective*. *Experimental and Therapeutic Medicine*. *In Press*.*)*, we considered the tumor-suppressive role of miR-132 in HCC to be well established.

In combination with our previous study *(Xin Zhang*, *et al*. *Down-regulation of MicroRNA-132 Indicates Progression in Hepatocellular Carcinoma*: *A Clinical Perspective*. *Experimental and Therapeutic Medicine*. *In Press*.*)*, our findings are consistent with those by Liu, et al.[15] and Wei, et al. At first, the down-regulation of miR‑132 in HCC can be considered well confirmed. Wei, et al. made use of only 20 cases, Liu, et al.[15] employed 40 pairs of tissues and 4 HCC cell lines, while in ours *(Xin Zhang*, *et al*. *Down-regulation of MicroRNA-132 Indicates Progression in Hepatocellular Carcinoma*: *A Clinical Perspective*. *Experimental and Therapeutic Medicine*. *In Press*.*)*, a more than doubled cohort of 95 pairs of tissues was considered. Next, the down-regulation of miR-132 correlates with several clinical parameters, which indicates the progression of HCC. Liu, et al [14] assumed miR-132 negetively correlated with tumor differentiation, TNM stage and lymph node metastasis, while in the previous research by us *(Xin Zhang*, *et al*. *Down-regulation of MicroRNA-132 Indicates Progression in Hepatocellular Carcinoma*: *A Clinical Perspective*. *Experimental and Therapeutic Medicine*. *In Press*.*)*, a doubled panel of parameters was taken into consideration such as distant metastasis, TNM stage, HBV-positive, nm23-expression, Ki-67 LI and tumor infiltration or no capsule. Lastly, multiple cellular functions are validated to be influenced by miR-132 in HCC, which Liu, et al.[15] verified with the single cell line of HepG2 and we later confirmed with the quadrupled scale of four HCC cell lines, namely HepG2, HepB3, SNU449 and SMMC-7221.

The natural language processing analysis captured 64,577 HCC-related records of PubMed titles and abstracts in total with 1800 HCC-related genes identified. GO analysis classified all the 1800 obtained genes in accordance with molecular function, cellular component and biological process. Among all the specific items with statistical significance (P< = 0.05), signal transduction activity (n = 384; P = 0.000166595) and nucleic acid binding activity (n = 327; P = 2.22E-10) stood out in molecular functions; among cellular components, nucleus (n = 536; P = 1.48E-10) shared the highest occurrence; in terms of biological processes, developmental processes definitely appeared the most prominent with an count of 531 (P = 1.23E-10). The above GO analysis results would equip researchers with better defined orientations as to molecular studies of HCC. Followed-up pathway analysis recognized 24 statistically significant HCC-related pathways with cytokine-cytokine receptor interaction (n = 111; P = 1.41E-17) and MAPK signaling pathway (n = 100; P = 3.92E-11) notably sharing the most occurrences. Lastly, network analysis was performed in an attempt to predict the potential gene regulatory/interacting networks. A network composed of multiple genes was established and it is worthwhile to mention that some of highly connected genes, which are known as hub genes, might play indispensably crucial role in the stabilization, interaction, and regulation of gene networks. Connectivity analysis pointed out the top connectivity of PIK3CA and PIK3R2. PI3Ks, or phosphatidylinositol 3-kinases, are a class of lipid kinases, which can phosphorylate the 3'OH end of the inositol ring of phosphoinositides and cope with a wide range of cellular activities, such as cell proliferation, migration and development. PIK3CA gene encodes a 110 kDa catalyzing subunit of p110α protein in human. The mutation of PIK3CA gene can result in the activation of protein kinase B signaling, which has been observed in multiple cancers. [44] PIK3R2 gene encodes the β isoform of the PI3K p85 regulatory subunit. PIK3R2 has been considered vital in some malignancies and reported as the target of several microRNAs, such as miR-126 in esophageal squamous cell carcinoma[45], miR-126-3p in hepatocellular carcinoma[46], and so forth. Quite interestingly, Liu, et al[15] looked for the potential miR-132 targets with the help of prediction algorithms such as TargetScan, miRanda, and miRWalk and later on considered PIK3R3 to be a potential target in HCC. Moreover, the following luciferase assay confirmed that PIK3R3 is a directly target of miR‑132. In a certain way, the finding of PIK3R3 as a direct target in a certain way proved the current study to be practical and promising for future usages, since in the NLP analysis the top two genes with the highest connectivity are PIK3CA and PIK3R2, both of which. Similar to PIK3R3, belong to regulatory subunits of phosphoinositide-3-kinase. Other notable hub genes have also been claimed to be associated with cancers, including MAPK1[47], MAPK3[48], JAK2[49], EGFR[50], KRAS[51] and NRAS[52]. Given the complexity of multi-step processes of carcinogenesis, development, progression and metastasis, we might suggest that researchers and practitioners should consider combining some of the above hub genes together or with well-established biomarkers to better predict the prognosis.

To enhance the sensitivity of miR-132 target gene prediction, a wide range of eleven bioinformatics tools were employed for the prediction process. For reliability/credibility control purpose, only when a certain target gene got named by at least four bioinformatics tools would it be included. Subsequently, 501 potential target genes turned out qualified. GO analysis for miR-132 predicted genes identified transcription regulatory activity (n = 15; P = 1.16E-06) and nucleic acid binding activity (n = 21; P = 2.25E-05) as the only two statistically significant items in molecular function (P< = 0.05); in the cellular component section, nucleus was the only statistically significant one with a count of 30 and a P-value of 5.98E-06; as to biological processes, among items with statistical significance, developmental processes (P = 1.13E-08) and RNA metabolism (P = 2.57E-05) stood out with primal top occurrences of 28 and 23 respectively. The above GO analysis might better illuminate miR-132-related research focus for scientists and clinical practitioners. Later, the pathway analysis established a total of 70 pathways with four proved statistically significant (P< = 0.05), namely neurotrophin signaling pathway (count = 5; P = 0.002082), MAPK signaling pathway (count = 5; P = 0.029881), VEGF signaling pathway (count = 3; P = 0.044476), and adherens junction (count = 3; P = 0.04664). Network analysis displayed the potential unique networks on how miR-132 interacts with other genes. In Fig 9, we used lines of different colors to differentiate the sources of connecting relationships. It could be seen that data from experiments took up the most followed by those from literature, with very few originated from databases. We believe that this way of display would help interested researchers evaluate the powers of network for further study purposes. Forthcoming connectivity analysis uncovered the highest connectivity of KRAS in the potential network of miR-132 predicted genes, with sixteen interacting genes, i.e. ARID1A, BRCA1, DNMT3A, EGR1, FOXO3, FRS2, GNA12, HMGA2, MAPK1, PTCH1, PXN, SGK3, SIRT1, USP9X, WT1 and YWHAG (z-test, P = 0.00618). KRAS gene encodes the protein GTPase KRas, also known as KRAS, whose normal form is a vital part in multiple signal transduction in normal tissue at early stage[53]. However, its mutation might be associated with some malignancies, especially colorectal cancer [54, 55]. MAP1 is the second highest connected hub genes, interacting with fifteen other genes (BRCA1, CITED2, DUSP9, EGR1, FOXO3, FRS2, GATA2, GNA12, KRAS, NET1, PEA15, PXN, SGK3, SPRY1, and YWHAG) (z-test, P = 0.001543) (Fig 10, S2 Table). There are two members, MAP1A and MAP1B, in the MAP1 (Microtubule-associated protein 1) family. Of relatively high molecular mass, both MAP1A and MAP1B are expressed mainly in the nervous system and associated with axon guidance and synaptic function [56, 57]. Considering the above, we speculated that KRAS and MAP1 might be partly responsible for the confirmed associations between miR-132 and several cancers, such as colorectal cancer[8], glioma[10] and primary glioblastoma multiforme[58]. An earlier study by Chang, et al.[59] has put forward the concept of applying gene expression profile of peripheral blood for the detection of colorectal cancer. Researchers interested can capitalize on methods mentioned[59] in combination with findings in the current research, namely, discovering whether the abovementioned hub genes, especially KRAS and MAP1, would be appropriate for blood-based detection assays in HCC.

At length, the analytical integration synthesized the overlaps with fifty-nine genes identified to be potentially both HCC- and miR-132-related (Table 5). Given the relatively scarce scale of genes spotted, only network analysis was conducted for further analysis (Fig 11). Four pathways stood out, i.e. adherens junction, VEGF signaling pathway, neurotrophin signaling pathway, and MAPK signaling pathway. Involved genes of these four pathways in the study are as follows: TCF7L2 might interact with miR-132 via adherens junction; PXN could be associated with miR-132 via VEGF signaling pathway; Neurotrophin signaling pathway could be in charge of the interactions between miR-132 and YWHAG, FOXO3 and FRS2; MAPK signaling pathway might mediate signaling and communicating between miR-132 and DUSP9, GNA12, MAPK1, NLK and KRAS. KRAS and MAPK1 are considered to be the most connected among the miR-132 predicted target genes in the integrated network. In our previous work *(Xin Zhang*, *et al*. *Down-regulation of MicroRNA-132 Indicates Progression in Hepatocellular Carcinoma*: *A Clinical Perspective*. *Experimental and Therapeutic Medicine*. *In Press*.*)*, several associations have been established with statistical significance between the expression of miR-132 and metastasis, clinical TNM stage, and tumor capsular infiltration, which might be mutually supported by four specific pathways in the overlapped network, namely adherens junction, VEGF signaling pathway, neurotrophin signaling pathway, and MAPK signaling pathway. Being multi-tasking, adherens junctions are among the most frequent class of intercellular adhesions and responsible for preserving cellular polarity and tissue structures, confining cell migration and proliferation, and generating drives for morphogenesis[60]. As can be observed from the *in vitro*, miR-132 can significantly inhibit HCC cell proliferation, which might be the very results via the pathway of adherens junctions. And the increased ability of colony formation as well as migration and invasion due to the loss of miR-132 [15] can be the very result of the dysregulation of the pathway of adherens junction. VEGF, or vascular endothelial growth factor, is a protein actively involved in human vasculogenesis and angiogenesis [61]. Accumulating evidence revealed that VEGFR-2, a crucial participant of VEGF-provoked reactions in endothelial cells, is vital to both physiologic and pathologic vasculogenesis and angiogenesis. A serial of different signaling pathways, including VEGF signaling pathway, are followed by the binding of VEGF to VEGFR-2, increasing the vascular permeability endothelial cells and moderating their proliferation and migration[62]. We speculate that the decreased viability of HCC cell lines by miR-132 in the in vitro experiments might be associated with the VEGF signaling pathway. So might the impaired proliferation rate of HCC cell lines. Likewise, VEGF signaling pathway might be also responsible for the negative correlations between miR-132 and TNM stage, lymph node metastasis, and tumor infiltration or no capsule reported formerly [15] *(Xin Zhang*, *et al*. *Down-regulation of MicroRNA-132 Indicates Progression in Hepatocellular Carcinoma*: *A Clinical Perspective*. *Experimental and Therapeutic Medicine*. *In Press*.*)*. Neurotrophins are a class of trophic molecules actively engaged in differentiation and survival of neurocytes. Neurotrophin signaling pathway interacts with an abundant range of intracellular signaling cascades, which might exert their essential influence on both the development of neurocytes and some of the higher-order behaviors like learning and memory. Neurotrophin signaling pathway and related miRNAs have been considered relevant to several cancers and brain diseases, which might provide potential novel diagnostic and therapeutic methods via the crosstalk between neurotrophins and miRNAs[63]. MAPK (mitogen-activated protein kinase) signaling pathway is an exceedingly conserved cascade involved in multiple human cell activities, such as proliferation, differentiation and migration. Though immature and still at early stage, recent study has showed that MAPK signaling pathway inhibitors promise to be impetus of target drugs due to the fact that around one third of cancers in human are influenced by mutations of MAPK signaling pathway[64]. The changes of viability and proliferation in HCC cells tranfected with miR-132 mimics/inhibitors have a great potential to do with the MAPK signaling pathway. Speculations are that three pathways, namely adherens junction, VEGF signaling pathway and MAPK signaling pathway, might be involved in significant association between HCC metastasis and miR-132 while adherens junction and MAPK signaling pathway have higher potentials to correlate with the tumor capsular infiltration of miR-132-related HCC. Clinical TNM stage, whose relationship with miR-132 has been proved statistically significant, is a comprehensive assessment scale with many factors taken into account. Thus, more efforts should be put into the possible pathways linked to the clinical TNM stage of HCC, which thereby would not be discussed here. Moreover, the decreased viability and lowered proliferation rate of HCC cells influenced might be very likely to have connections with the three pathways as well. Nevertheless, pathways related to the accelerated apoptosis from the *in vitro* are not included in those established by the analytical integration.

Inspired by Gao, et al.[65], the current study has explored gene signatures for HCC, miR-132 predicted target genes and the corresponding overlaps and presented a number of comprehensive analyses with the assistance of bioinformatics technology. There are several features of the study that excels itself among all the others. First of all, the formerly reported tumor-suppressive role of miR-132 have been further confirmed by *in vitro*, and as far as we are concerned, the study is the first one to elucidate the gene signatures for either HCC or miR-132 predicted target genes, not to mention the corresponding overlaps. Then, it is also worth commending that the NLP analysis of HCC, miR-132 predicted target genes and the relevant analyses (GO analysis, pathway analysis, network analysis and connectivity analysis) are reusable data which may prove helpful to the forthcoming researches. Furthermore, the current study provides potential molecular basis for the down-regulation of miR-132 in HCC, complementing the shortcomings of our previous work *(Xin Zhang*, *et al*. *Down-regulation of MicroRNA-132 Indicates Progression in Hepatocellular Carcinoma*: *A Clinical Perspective*. *Experimental and Therapeutic Medicine*. *In Press*.*)*. Likewise, the potential mechanisms can be also used for the explanation of the established, significant associations between the expression of miR-132 and several clinical parameters. Last but not least, the nearly quadrupled union of eleven bioinformatics tools adds the unprecedented reliability and credibility to the prediction of potential miR-132 target genes, while, to our knowledge, all the similar works[65–67] regarding other microRNAs or other cancers done previously adopted only a panel of three bioinformatics tools at most. Nevertheless, limitations still exist with a primal one standing out that either NLP analysis of HCC or prediction of miR-132 target genes, to a considerable extent, are based on bioinformatics techniques instead of experiments, which might cause false positive results. Hence, our research team has hereby decided to select some of hub genes and pathways for combined detections with other biomarkers in clinical practice and further *in vitro* experiments in the future.

## 5. Conclusion

To summarize, the study has further confirmed the previously reported tumor-suppressive role of miR-132 in HCC by *in vitro* and encapsulated gene signatures for HCC, miR-132 predicted target genes and the corresponding overlaps, which complements the previously-found tumor-suppressive role of miR-132 in HCC. A full panel of NLP analysis of HCC, prediction of miR-132 target genes, comprehensive analyses and analytical integration was completed, providing us with unprecedentedly illuminated insights into the underlying molecular mechanisms of miR-132 in HCC. It is also suggested that miR-132 and its potential targets might prove to be effective for diagnosis, individualized treatments and prognosis of HCC patients. However, due to the limitations of bioinformatics technology, combined detections of miR-132 with other biomarkers in clinical practice and further *in vitro* experiments are still pressingly demanded, which the team decides to focus on in the future.

## Supporting Information

S1 Table All the 70 pathways identified for miR-132 predicted target genes. Pathway analysis identified a total of 70 pathways for miR-132 predicted target genes.
(PDF)

S2 Table Listed results of connectivity analysis for miR-132 predicted target genes. Connectivity analysis was employed to display the interacting degrees of miR-132 predicted target genes.
(PDF)
