# Supplementary material for: An Encapsulation of Gene Signatures for Hepatocellular Carcinoma, MicroRNA-132 Predicted Target Genes and the Corresponding Overlaps
Source: PLoS One. 2016 Jul 28;11(7):e0159498. doi: 10.1371/journal.pone.0159498 (PMC4965135; doi:10.1371/journal.pone.0159498)
Supplement: S1 Table — Pathway analysis identified a total of 70 pathways for miR-132 predicted target genes. (PDF) [file pone.0159498.s001.pdf]

Term	Count	pvalue	genes
hsa05213:Endometrial cancer	4	0.001546349	MAPK1, KRAS, FOXO3, TCF7L2
hsa04722:Neurotrophin signaling pathway	5	0.002082375	MAPK1, YWHAG, KRAS, FOXO3, FRS2
hsa05216:Thyroid cancer	3	0.007377745	MAPK1, KRAS, TCF7L2
hsa05223:Non-small cell lung cancer	3	0.024284665	MAPK1, KRAS, FOXO3
hsa05221:Acute myeloid leukemia	3	0.027747732	MAPK1, KRAS, TCF7L2
hsa04010:MAPK signaling pathway	5	0.029881036	MAPK1, KRAS, NLK, GNA12, DUSP9
hsa04730:Long-term depression	3	0.03821865	MAPK1, KRAS, GNA12
hsa05211:Renal cell carcinoma	3	0.039235898	MAPK1, KRAS, SLC2A1
hsa04370:VEGF signaling pathway	3	0.044476344	MAPK1, KRAS, PXN
hsa04520:Adherens junction	3	0.046642537	MAPK1, NLK, TCF7L2
hsa04062:Chemokine signaling pathway	4	0.050478563	MAPK1, KRAS, FOXO3, PXN
hsa05210:Colorectal cancer	3	0.054524281	MAPK1, KRAS, TCF7L2
hsa05200:Pathways in cancer	5	0.05687468	MAPK1, KRAS, SLC2A1, PTCH1, TCF7L2
hsa05215:Prostate cancer	3	0.060426024	MAPK1, KRAS, TCF7L2
hsa04810:Regulation of actin cytoskeleton	4	0.070848046	MAPK1, KRAS, GNA12, PXN
hsa04916:Melanogenesis	3	0.072856961	MAPK1, KRAS, TCF7L2
hsa04320:Dorso-ventral axis formation	2	0.107387848	MAPK1, KRAS
hsa05020:Prion diseases	2	0.147175876	EGR1, MAPK1
hsa04960:Aldosterone-regulated sodium reabsorption	2	0.170228311	MAPK1, KRAS
hsa05219:Bladder cancer	2	0.174011964	MAPK1, KRAS
hsa05217:Basal cell carcinoma	2	0.221722158	PTCH1, TCF7L2
hsa04510:Focal adhesion	3	0.230021715	MAPK1, TLN2, PXN
hsa05214:Glioma	2	0.24975968	MAPK1, KRAS
hsa04920:Adipocytokine signaling pathway	2	0.263413549	SLC2A1, ACSL4
hsa04720:Long-term potentiation	2	0.266789693	MAPK1, KRAS
hsa05218:Melanoma	2	0.276829543	MAPK1, KRAS
hsa05212:Pancreatic cancer	2	0.280146839	MAPK1, KRAS
hsa05220:Chronic myeloid leukemia	2	0.29001162	MAPK1, KRAS
hsa04662:B cell receptor signaling pathway	2	0.29001162	MAPK1, KRAS
hsa04664:Fc epsilon RI signaling pathway	2	0.299747016	MAPK1, KRAS
hsa04914:Progesterone-mediated oocyte maturation	2	0.325087787	MAPK1, KRAS
hsa04012:ErbB signaling pathway	2	0.328193004	MAPK1, KRAS
hsa04540:Gap junction	2	0.33436249	MAPK1, KRAS
hsa04912:GnRH signaling pathway	2	0.361460801	MAPK1, KRAS

Term	Count	pvalue	genes
hsa04660:T cell receptor signaling pathway	2	0.390331961	MAPK1, KRAS
hsa04114:Oocyte meiosis	2	0.395954371	MAPK1, YWHAG
hsa04270:Vascular smooth muscle contraction	2	0.401527165	MAPK1, GNA12
hsa04110:Cell cycle	2	0.436569498	YWHAG, TTK
hsa04360:Axon guidance	2	0.446950876	MAPK1, KRAS
hsa04650:Natural killer cell mediated cytotoxicity	2	0.457149144	MAPK1, KRAS
hsa04910:Insulin signaling pathway	2	0.462180577	MAPK1, KRAS
hsa04310:Wnt signaling pathway	2	0.500856931	NLK, TCF7L2
hsa00360:Phenylalanine metabolism	1	1	MAOA
hsa00380:Tryptophan metabolism	1	1	MAOA
hsa00531:Glycosaminoglycan degradation	1	1	IDS
hsa00071:Fatty acid metabolism	1	1	ACSL4
hsa04666:Fc gamma R-mediated phagocytosis	1	1	MAPK1
hsa04142:Lysosome	1	1	IDS
hsa04350:TGF-beta signaling pathway	1	1	MAPK1
hsa03320:PPAR signaling pathway	1	1	ACSL4
hsa00350:Tyrosine metabolism	1	1	MAOA
hsa00330:Arginine and proline metabolism	1	1	MAOA
hsa00260:Glycine, serine and threonine metabolism	1	1	MAOA
hsa05412:Arrhythmogenic right ventricular cardiomyopathy	1	1	TCF7L2
hsa04120:Ubiquitin mediated proteolysis	1	1	BRCA1
hsa04340:Hedgehog signaling pathway	1	1	PTCH1
hsa00340:Histidine metabolism	1	1	MAOA
hsa05016:Huntington's disease	1	1	SOD2
hsa04622:RIG-I-like receptor signaling pathway	1	1	CYLD
hsa00270:Cysteine and methionine metabolism	1	1	DNMT3A
hsa05010:Alzheimer's disease	1	1	MAPK1
hsa04930:Type II diabetes mellitus	1	1	MAPK1
hsa04620:Toll-like receptor signaling pathway	1	1	MAPK1
hsa04150:mTOR signaling pathway	1	1	MAPK1
hsa00982:Drug metabolism	1	1	MAOA
hsa04530:Tight junction	1	1	KRAS
hsa04115:p53 signaling pathway	1	1	CCNG1
hsa04670:Leukocyte transendothelial migration	1	1	PXN

Term	Count	pvalue	genes
hsa04621:NOD-like receptor signaling pathway	1	1	MAPK1
hsa04630:Jak-STAT signaling pathway	1	1	SPRY1
