# Supplementary material for: An Encapsulation of Gene Signatures for Hepatocellular Carcinoma, MicroRNA-132 Predicted Target Genes and the Corresponding Overlaps
Source: PLoS One. 2016 Jul 28;11(7):e0159498. doi: 10.1371/journal.pone.0159498 (PMC4965135; doi:10.1371/journal.pone.0159498)
Supplement: S2 Table — Connectivity analysis was employed to display the interacting degrees of miR-132 predicted target genes. (PDF) [file pone.0159498.s002.pdf]

gene	degrees	p-value	interactions
ACSL4	1	0.800495457	RTN4
ARID1A	3	0.617984064	KRAS, MIB1, SOX2
BRCA1	7	0.215871313	FOXO3, KRAS, MAPK1, NFE2L2, PEA15, SIRT1, WT1
BTG2	2	0.71626834	CCNG1, EGR1
CCNG1	3	0.617984064	BTG2, FOXO3, PTCH1
CITED2	2	0.71626834	MAPK1, WT1
DNMT3A	3	0.617984064	KRAS, SOX2, WT1
DUSP9	1	0.800495457	MAPK1
EGR1	9	0.091855553	BTG2, FOXO3, GATA2, KRAS, MAOA, MAPK1, PTCH1, SOD2, WT1
FOXO3	11	0.030560662	BRCA1, CCNG1, EGR1, KRAS, MAPK1, NLK, SGK3, SIRT1, SOD2, WT1, YWHAG
FRS2	3	0.617984064	KRAS, MAPK1, SPRY1
GATA2	6	0.303412005	EGR1, MAPK1, SGK3, SOX2, TCF7L2, WT1
GNA12	2	0.71626834	KRAS, MAPK1
HMGA2	4	0.511404034	KRAS, LIN28B, SOX2, ZEB2
KRAS	16	0.000617607	ARID1A, BRCA1, DNMT3A, EGR1, FOXO3, FRS2, GNA12, HMGA2, MAPK1, PTCH1, PXN, SGK3, SIRT1, USP9X, WT1, YWHAG
LIN28B	2	0.71626834	HMGA2, SOX2
MAOA	1	0.800495457	EGR1
MAPK1	15	0.001543093	BRCA1, CITED2, DUSP9, EGR1, FOXO3, FRS2, GATA2, GNA12, KRAS, NET1, PEA15, PXN, SGK3, SPRY1, YWHAG
MIB1	2	0.71626834	ARID1A, USP9X
NET1	1	0.800495457	MAPK1
NFE2L2	3	0.617984064	BRCA1, SIRT1, SOD2
NLK	2	0.71626834	FOXO3, TCF7L2
PEA15	2	0.71626834	BRCA1, MAPK1
PTCH1	4	0.511404034	CCNG1, EGR1, KRAS, WT1
PXN	3	0.617984064	KRAS, MAPK1, TLN2
RTN4	1	0.800495457	ACSL4
SGK3	5	0.403998308	FOXO3, GATA2, KRAS, MAPK1, SOD2
SIRT1	6	0.303412005	BRCA1, FOXO3, KRAS, NFE2L2, SOD2, YWHAG
SOD2	5	0.403998308	EGR1, FOXO3, NFE2L2, SGK3, SIRT1
SOX2	7	0.215871313	ARID1A, DNMT3A, GATA2, HMGA2, LIN28B, SOX6, ZEB2
SOX6	1	0.800495457	SOX2
SPRY1	3	0.617984064	FRS2, MAPK1, WT1
TCF7L2	2	0.71626834	GATA2, NLK
TLN2	1	0.800495457	PXN

gene	degrees	p-value	interactions
USP9X	2	0.71626834	KRAS, MIB1
WT1	9	0.091855553	BRCA1, CITED2, DNMT3A, EGR1, FOXO3, GATA2, KRAS, PTCH1, SPRY1
YWHAG	4	0.511404034	FOXO3, KRAS, MAPK1, SIRT1
ZEB2	2	0.71626834	HMGA2, SOX2
ZEB2	2	0.725056126	HMGA2, SOX2
